# Prevalence of Comorbidities and Baseline Characteristics of LAP-BAND AP® Subjects in the Helping Evaluate Reduction in Obesity (HERO) Study

**DOI:** 10.1371/journal.pone.0078971

**Published:** 2013-11-15

**Authors:** Nancy Dreyer, John B. Dixon, Ted Okerson, Eric A. Finkelstein, Denise Globe

**Affiliations:** 1 Quintiles Outcome, Cambridge, Massachusetts, United States of America; 2 Monash University and the Baker Heart and Diabetes Institute, Melbourne, Australia; 3 Medical Affairs, Allergan, Inc., Irvine, California, United States of America; 4 University of California Irvine, Irvine, California, United States of America; 5 Duke-NUS Graduate Medical School, Singapore, Singapore; 6 Global Health Outcomes Strategy & Research, Allergan, Inc., Irvine, California, United States of America; University of Texas Health Science Center at San Antonio, United States of America

## Abstract

**Objective:**

To describe the baseline characteristics in patients who chose placement of a LAP-BAND AP® System (LBAP) and participated in the Helping Evaluate Reduction in Obesity (HERO) Study across regions.

**Patients and Methods:**

HERO is a five- year, prospective, multicenter, international study of patients with LBAP placement between July 22, 2009 and January 31, 2011. In addition to baseline and peri-surgery clinical data, seven follow up visits are scheduled at 3, 6 and 12 months, and annually through year five. Data collection included family and medical history, clinical outcomes, laboratory data, health-related quality of life (HRQoL), productivity, healthcare resource utilization, and adverse events.

**Results:**

LBAP were placed in 1106 enrolled patients; 56.6% from the US, 26.3% from Europe, 7.1% from Canada, and 10.0% from Australia. The majority were female (n = 877 (79.3%)) with a mean age of 43 years (s.d. = 11.4) and mean body mass index of 45.1 kg/m^2^ (s.d. = 6.9). The most common comorbidities were hypertension (HTN) (overall  = 42.9%) and diabetes (overall 22.2%, with 27% from the US and 14% from Europe). Overall, less than 5% had a history of cardiovascular disease. The prevalence rates of HTN, diabetes and cardiovascular disease were significantly (p<0.001) higher in men than in women across all regions. Overall HRQoL also worsened with increasing BMI.

**Conclusions:**

The HERO study is the first large, multinational and long-term registry with the LBAP. This study will provide real-world outcomes data on LAGB that will help inform patient choice, clinician treatment strategies, and payer reimbursement decisions.

## Introduction

Obesity is a global public health problem of epidemic proportions, affecting 205 million men and 297 million women over the age of 20 worldwide in 2008 [1]. Obesity has a profound impact on increased risk for developing comorbid chronic diseases and premature mortality [2], [3]. Excess weight also imposes an economic burden on individuals and health care systems, including direct costs from health resource utilization and indirect societal costs from absenteeism and workplace injuries, disability payments, and decreased productivity [4]–[8].

Due to the notable clinical, economic and humanistic impact of obesity, weight reduction is a critical goal for both patients and clinicians. Bariatric surgery is one such weight reduction option for a subset of obese individuals. The choice by patients and their physicians to use a surgical option for weight loss is influenced by various factors including weight, comorbidity, gender, age, geography, safety and reimbursement. Bariatric surgery typically results in greater and more sustainable weight loss compared to non-surgical approaches [9]. In addition, a growing body of evidence suggests that modest sustained weight loss achieved through bariatric surgery can improve health outcomes, including improving co-morbid conditions, in surgery-eligible obese individuals more successfully than diet, exercise, and/or medications [10]. As a result, multiple professional organizations including the American Diabetes Association, the American Association of Clinical Endocrinologists and governmental health agencies such as the National Institute for Clinical Excellence (NICE) recommend bariatric surgery as an option for adults with Body Mass Index (BMI) ≥40 kg/m^2^ or (BMI) >35 kg/m^2^ with one or more comorbid conditions [11], [12], [13], [14] and for any patient with a BMI ≥40 kg/m^2^ [12], [13]. Bariatric procedures are now mostly done laparoscopically and have widely-acceptable safety profiles, with adjustable gastric bands typically being recognized as the safest of available procedures [15]–[17].

Gastric banding was first used in the early 1990's. The LBAP, typically placed laparoscopically, is the latest generation of adjustable gastric banding systems, with improvements designed to enhance the safety and efficacy of the device [18]. In the United States (US), these new generation adjustable gastric bands were approved in 2006 for use in patients with a BMI of 40 kg/m^2^, or 35 or greater with an existing comorbidity; similar approvals for other countries in the study include Canada in 2006, the EU in 2005, and Australia in 2003. In 2011, the Food and Drug Administration (FDA) approved an expanded the indication for the LBAP, lowering the BMI restriction to include adults with BMI over 30 kg/m^2^ who have at least one obesity-related comorbid condition, such as diabetes or heart disease [18].

Given the many options available and limited duration of randomized trials, there is growing interest in examining the long-term safety and other outcomes for those who undergo bariatric surgery, including LBAP. These outcomes extend beyond weight loss to include changes in obesity-related medical conditions, health resource utilization, and health-related quality of life (HRQoL). With respect to the LBAP, however, there are also gaps in knowledge about the long-term benefits and risks of this device when used with the latest surgical techniques in a more heterogeneous patient population. Although the literature summarizes adverse events and the impact of banding on weight loss, many of these citations are case studies or single site convenience samples, and may use older bands or obsolete surgical techniques [19]–[21].

To provide more current and relevant information to decision makers, the Helping Evaluate Reduction in Obesity (HERO) Study (NCT00953173) is designed to provide real-world effectiveness and safety evidence of the LBAP. The objectives of this manuscript are to provide a detailed description of the HERO study design and patient characteristics in the participating regions.

## Methods

The HERO Study was designed to: (1) evaluate outcomes among LBAP recipients in a real world setting; (2) describe the prevalence of and/or changes from baseline comorbidities after placement of the LBAP; (3) assess changes in HRQoL measures; (4) quantify changes in healthcare expenditures; and (5) monitor the long term safety of the device by summarizing Adverse Events (AEs).

### Study Design

The HERO study screened 1,153 patients from 29 sites. Sequential enrollment, an accepted hallmark of quality for registries, [22] was used to reduce the potential for bias associated with selective enrollment. The study was designed to incorporate multi-country experience, with physicians and patients from Australia (AUS), Canada (CA), Europe or EU countries (Belgium, Italy, the UK), and the US. The decision to have the LPAP procedure was made independently of this study and prior to patient enrollment in this study. Patients provided written informed consent before enrolling into the HERO study. The study was approved by the Ethics Committees and Institutional Review Boards appropriate for each country. They included:

- Calvary Health Care Adelaide (CHCA) Human Research Ethics Committee.
- Melbourne Clinic Research Ethics Committee.
- Commissie Medische Ethiek UZ Brussel.
- Aizenda USL 11 Empoli.
- Universita degli studi di Napoli “Federico II” Comitato Etico per le Attivita Biomediche (Ethics Committee for the Biomedical Activities) “Carlo Romano”.
- Comitato Etico per la Sperimentazione Clinica della Provincia di Vincenza.
- Birmingham, East, North, and Solihull Research Ethics Committee.
- Sussex NHS Research Consortium.
- Derby Hospitals NHS Foundation Trust.
- Quorum IRB.
- IRB of Regional West Medical Center.
- BRANY IRB.
- Scripps IRB.

Inclusion and exclusion criteria are consistent with the original product labeling in the participating countries (i.e., prior to the lower BMI approval in 2011) such that adults with a BMI ≥40 kg/m^2^ or a BMI ≥35 kg/m^2^ with one or more severe comorbid conditions were included in the study. Patients were excluded if they had Type 1 diabetes or had undergone prior bariatric surgery.

### Data Collection

Study data are collected pre-operatively (no more than 8 weeks prior to surgery), peri-operatively, and post-operatively at 3, 6 and 12 months (±4 weeks), and at 2, 3, 4, and 5 years (±8 weeks). These data collection visits are scheduled to comport with routine care and to allow for the flexibility in scheduling that is common to observational research. In the event that a patient misses a follow-up visit, a site coordinator will phone the patient to capture key safety data. If the site coordinator is unable to reach the patient after three attempts at different times of the day, a registered letter (i.e., requiring signature as proof of delivery) will be sent to the patient in an effort to obtain key safety data. The target retention rate is 70% or better at five years.

All data are collected via a secure web-based data entry system (Outcome System®, Quintiles Outcome, Cambridge, MA USA). An electronic case report form (eCRF) is completed for each study visit and is stored in a secure server in compliance with FDA 21 CFR Part 11 guidelines. Regular study monitoring is conducted, with a target of achieving nearly 100% source data verification. De-identified data are transferred via a secure SharePoint site to the sponsor for data analyses.

### Measurements of HERO Study

Medical chart reviews of demographic information such as age, gender, education level, and employment status, family and medical history were collected at baseline. Concomitant medication usage was retrieved from medical charts at baseline and will be collected at each scheduled visit. Weight, height, and waist circumference were measured at baseline and are obtained at each of the scheduled physician visits.

Peri-surgery data included procedure details, device information (serial number), and any adverse events.

Laboratory evaluations which included hemoglobin A_1c_ (HbA_1c_), fasting glucose, fasting lipid profile (total cholesterol, high density lipoprotein [HDL], low density lipoprotein [LDL], and triglycerides were collected at baseline and will be collected at yearly follow up visits.

Patient reported outcomes, collected at baseline and at each scheduled follow-up visit, are used to assess HRQoL and work productivity. These instruments included the Short Form-12 (SF-12®) [23], the European Quality of Life –5 Dimensions (EQ-5D^TM^) [24], the Impact of Weight on Quality of Life – Lite (IWQOL-Lite) [25], and the Work Productivity and Activity Impairment Questionnaire General Health version 2.0 (WPAI) [26], all of which have been validated.

#### SF-12

The 12-Item Short Form Health Survey is a general health status measure. It is self-administered and assesses 8 domains: physical functioning, role limitations due to physical health, bodily pain, general health perceptions, vitality, social functioning, role limitations due to emotional problems, and mental health. Summary Mental and Physical Component scores are generated using a proprietary algorithm supplied by the instrument developers. A 4-week recall period is used and the scoring method used normalized responses to 50±10, with higher scores indicating better health status [23].

#### EuroQol (EQ-5D)

The EQ-5D is a generic health-related quality of life measure. It is self-administered and has 5 dimensions assessing mobility, self-care, usual activities, pain/discomfort, anxiety/depression. It also includes a visual analog scale that asks respondents to rate their current health from 0–100, with 0 representing death and 100 indicating perfect health. The 5-item questionnaire can also be transformed to a societal-based utility score, ranging from 0–1, with higher scores reflecting better health status, as recommended by its authors [24].

#### Impact of Weight on Quality of Life – Lite (IWQOL-Lite)

The IWQOL-Lite is the shortened version of IWQOL, an obesity-specific QOL instrument consisting of 31 items and 5 domains: Physical Function (11 items), Self-Esteem (7 items), Sexual Life (4 items), Public Distress (5 items), and Work (4 items). A domain score can be calculated by summing all items within that domain and subsequent transformation to a score between 0 and 100. A total score can be obtained by summing scores for all 31 items. Lower scores indicate a better QOL [25].

#### Work Productivity and Activity Impairment (WPAI)

The WPAI yields scores for four domains: Absenteeism (work time missed) with number of hours missed from work because of their health problems, presenteeism (impairment at work/reduced on-the-job effectiveness) or how much did health problems affect productivity while working, work productivity loss (overall work impairment/absenteeism plus presenteeism), and activity impairment (non-work related). WPAI outcomes are expressed as impairment percentages, with higher numbers indicating worse outcomes [26].

Changes in healthcare expenditures, such as indirect cost, over time will be derived using the WPAI scores to estimate cost to employers based on amount of work days missed. In addition, changes in cost from any decreased use of medications for hypertension and/or diabetes medication can be estimated by reported changes in use of anti-hypertensive and anti-diabetic medications at baseline vs. follow up visits.

Food intake, eating patterns, target weight, and satisfaction with weight loss and with the benefits provided by LBAP, are also measured at baseline and annually.

Throughout the study, AEs are captured by asking patients about any device related complications that may have occurred and by investigators' evaluation at each annual visit.

Information about AEs and rates of band explants will be used to help establish its risk-benefit profile.

### Descriptions of Baseline Variables Used in the HERO Study

Duration of obesity (in years) was computed as the difference in years between current age and age when patients became obese (BMI >30). Presence of current conditions of T2DM, HTN, or hyperlipidemia was separately coded. Subjects who had either myocardial infarction, atrial fibrillation, peripheral vascular disease, congestive heart failure or stroke in their medical history were categorized as positive for cardiovascular disease. Duration of T2DM, HTN, or any one cardiovascular disease in years was computed as the difference between the LBAP implantation date and the reported disease diagnosis date.

Proportion of anti-diabetes medication utilization was categorized as insulin, oral medication, diet or no treatment. Use of anti-hypertensive medication was binary coded. Proportions of subjects treated with 1, 2 or more lipid lowering agents were also reported.

### Statistical Analysis

Descriptive statistics were calculated for demographic, comorbid conditions, medication usage, and obesity measures, with basic statistics given for the continuous data (number, mean, and standard deviation), by regions. Regions were categorized as follows: US, CA, AUS, and EU (UK, Italy, and Belgium). Analyses were performed using SAS, Version 9.2 [27].

## Results

Patient recruitment started in July 2009, and 1,153 patients were screened for eligibility by January 31, 2011. There were 47 screen failures (Table 1). HERO was fully enrolled on January 31, 2011 with 1,106 patients implanted with LBAP; 626 (56.6%) from US sites and 291 (26.3%) from European sites, 79 (7.1%) from Canadian sites, and 110 (10%) from Australia sites.

**Table 1:** Patient Disposition, by Region.

	Total	US	Europe^a^	Canada	Australia
Patients screened for eligibility	1153	644	300	80	129
**Screening failures**	**18**	**3**	**7**	**0**	**8**
Patient did not meet BMI eligibility criteria	7 (38.9%)	1 (33.3%)	4 (57.1%)	0 (0.0%)	2 (25.0%)
No expectation of compliance with the study plan	2 (11.1%)	1 (33.3%)	1 (14.3%)	0 (0.0%)	0 (0.0%)
Patient had prior bariatric surgery	1 (5.6%)	1 (33.3%)	0 (0.0%)	0 (0.0%)	0 (0.0%)
Patient did not provide IC	8 (44.4%)	0 (0.0%)	2 (28.6%)	0 (0.0%)	6 (75.0%)
**Withdrew prior to baseline**	**12**	**1**	**0**	**0**	**11**
Subject withdrew consent	5 (41.7%)	0 (0.0%)	0 (0.0%)	0 (0.0%)	5 (45.5%)
Subject did not undergo the surgery to implant LAP-BAND AP® System	2 (16.7%)	1 (100.0%)	0 (0.0%)	0 (0.0%)	1 (9.1%)
Subject did not comply with the ICF/CIP requirements	1 (8.3%)	0 (0.0%)	0 (0.0%)	0 (0.0%)	1 (9.1%)
Other	4 (33.3%)	0 (0.0%)	0 (0.0%)	0 (0.0%)	4 (36.4%)
**Withdrew prior to surgery**	**17**	**14**	**2**	**1**	**0**
Subject did not undergo the surgery to implant LAP-BANDAP® System	16(94.1%)	13(92.9%)	2 (100.0%)	1 (100.0%)	0 (0.0%)
Unsuccessful LAP-BAND AP® System implantation	1 (5.9%)	1 (7.1%)	0 (0.0%)	0 (0.0%)	0 (0.0%)
**Patients undergoing surgery for** **LAP-BAND AP®**	**1106**	**626**	**291**	**79**	**110**

a European countries included Belgium, Italy and UK.

As seen in Table 2, the study population (n = 1,106) is predominantly female (79%), with a mean age of 43 years ±11.4 (44–45 for US/CA and 41 for EU/AU). More than half (56%) of the cohort have completed high school and over a third (36%) have a university degree. At the time of study enrollment, 61% of patients were employed full-time and 61% were married. A history of parental obesity was reported by 57% of the overall cohort. The mean BMI at baseline was 45.1 kg/m^2^ ±6.9, and mean waist circumference of 126.2 cm ±16.8. On average, patients were obese for 18 years ±10.7 prior to implantation.

**Table 2:** Key Demographic Variables by Region.

Demographics	Overall (N = 1106)	US (N = 626)	Europe^a^ (N = 291)	Canada (N = 79)	Australia (N = 110)
Age, years Mean (sd)	43.1 (11.4)	43.9 (11.4)	41.5 (11.4)	45.1 (10.9)	41.7 (11.7)
Gender, n (%)					
Female	877 (79%)	499 (80%)	230 (79%)	64 (81%)	84 (76%)
Male	229 (21%)	127 (20%)	61 (21%)	15 (19%)	26 (24%)
Education					
Less than High School	86 (8%)	1 (0%)	78 (27%)	2 (3%)	5 (5%)
High School Diploma	616 (56%)	360 (58%)	159 (55%)	29 (37%)	68 (62%)
University degree	401 (36%)	265 (42%)	51 (18%)	48 (61%)	37 (34%)
No Response	3	0	3	0	0
Employment status					
Full-time	653 (61%)	414 (67%)	131 (49%)	52 (66%)	56 (51%)
Part-time	111 (10%)	41 (7%)	41 (15%)	6 (8%)	23 (21%)
Freelancer	39 (4%)	13 (2%)	17 (6%)	5 (6%)	4 (4%)
Currently not working	270 (25%)	151 (24%)	76 (29%)	16 (20%)	27 (25%)
No Response	33	7	26	0	0
Marital status					
Married	672 (61%)	374 (61%)	158 (54%)	45 (57%)	68 (62%)
Parental history of obesity					
Yes	610 (57%)	374 (61%)	146 (54%)	32 (42%)	58 (55%)
No	454 (43%)	238 (39%)	124 (46%)	45 (58%)	47 (45%)
No Response	42	14	21	2	5
BMI (kg/m^2^)					
Mean (sd)	45.1 (6.9)	45.4 (7.0)	45.2 (6.9)	45.1 (6.8)	43.6 (5.6)
Waist circumference, cm					
Mean, sd	126.2 (16.8)	125.9 (17.8)	126.7 (16.8)	126.1 (14.1)	127.0 (13.3)
No response	5	3	2	0	0
Duration of obesity, years					
Mean, sd	18.2 (10.7)	18.7 (10.8)	17.3 (10.3)	21.2 (11.4)	15.3 (9.4)
Unknown	230	112	73	7	38
Age became obese, years					
Mean, sd	25.2 (11.8)	25.5 (12.1)	24.2 (11.0)	24.0 (11.2)	27.6 (12.1)
Unknown	230	112	73	7	38

a European countries included Belgium, Italy and UK.

Regional differences in comorbidities at baseline were observed (Table 3). The most common comorbidity was HTN (43%). One in 2 obese patients had HTN in the US as compared with 1 in 3 in EU, CA, or AUS. The mean duration since HTN diagnosis was the longest for the US obese patients (8.2 years) as compared with EU (6.9 years), CA (7.3 years) or AUS (7.5 years). The majority (83%) of patients took hypertensive medications.

**Table 3:** Baseline Comorbid Conditions by Region.

	Overall (N = 1106)	US (N = 626)	Europe^a^ (N = 291)	Canada (N = 79)	Australia (N = 110)
Hypertension, N (%)	474 (42.9%)	310 (49.5%)	99 (34.0%)	31 (39.2%)	34 (30.9%)
Number of years with hypertension, Mean (sd)	7.7 (7.6)	8.2 (8.1)	6.9 (5.9)	7.3 (5.7)	6.1 (7.5)
Any Hypertension Medication Usage, N (%)	391 (82.5%)	259 (83.5%)	74 (74.7%)	30 (96.8%)	28 (82.4%)
Type 2 Diabetes, N (%)	245 (22.2%)	169 (27.0%)	41 (14.1%)	18 (22.8%)	17 (15.5%)
Number of years with Type 2 Diabetes, mean (sd)					
Overall	7.0 (6.2)	6.9 (6.4)	8.5 (4.9)	7.6 (7.1)	3.9 (3.1)
Patients treated with insulin	11.7 (6.9)	11.5 (7.5)	12.9 (3.9)	16.1 (7.4)	7.1 (3.9)
Patients treated without insulin	5.5 (4.8)	5.3 (5.1)	7.3 (4.3)	5.1 (4.9)	2.8 (1.9)
Anti-Diabetic Medication Usage^b^, N (%)					
Treated with Insulin	65 (26.5%)	47 (27.8%)	10 (24.4%)	4 (22.2%)	4 (23.5%)
Treated with oral medication	186 (75.9%)	125 (74.0%)	35 (85.4%)	12 (66.7%)	14 (82.4%)
Treated with diet	84 (34.3%)	60 (35.5%)	4 (9.8%)	12 (66.7%)	8 (47.1%)
Without treatment	7 (2.9%)	6 (3.6%)	1 (2.4%)	0 (0.0%)	0 (0.0%)
Unknown	1 (0.4%)	1 (0.6%)	0 (0.0%)	0 (0.0%)	0 (0.0%)
Cardiovascular Disease, N (%)	49 (4.4%)	33 (5.3%)	8 (2.7%)	5 (6.3%)	3 (2.7%)
Number of years with any one of the cardiovascular disease, Mean (sd)	6.2 (10.1)	6.5 (11.1)	4.1 (5.0)	4.9 (5.7)	9.6 (16.0)
Hyperlipidemia treated with lipid lowering agents, N (%)					
1 Med	200 (18.1%)	124 (19.8%)	32 (11.0%)	22 (27.8%)	22 (20.0%)
≥2 Meds	29 (2.6%)	25 (4.0%)	2 (0.7%)	2 (2.5%)	0 (0.0%)
Total	229 (20.7%)	149 (23.8%)	34 (11.7%)	24 (30.3%)	22 (20.0%)

a European countries included Belgium, Italy and UK.

b Patients may be on multiple anti-diabetic medications. The total proportion will not sum up to 100%.

Nearly a quarter of the cohort (22.2%) had T2DM (27% in the US, 14.1% in EU, 22.8% in CA, and 15.5% in AUS). Although diabetes was more prevalent in the US sample, European obese patients reported having had diabetes longer (mean 8.5 years) than their counterparts in the US (mean 6.9 years), CA (mean 7.6 years) or AUS (mean 3.9 years). In addition, the mean duration of diabetes history among patients taking insulin therapy was longer than those not taking insulin therapy (11.7 years versus 5.5 years). Overall, 27% of the diabetes cohort was treated with insulin. Three-quarters of the cohort took oral medications for T2DM, with higher proportion of EU patients taking oral medications (84.5%). Dietary monitoring was commonly practiced in CA (67%) but less so in EU (9.8%).

In general, the prevalence of cardiovascular disease (CVD) was low (4.4% overall) (Table 3). The mean duration of any one CVD was 6.2 years, with the longest duration in AUS (9.6 years).

One in five obese patients reported having hyperlipidemia, with the smallest proportion in EU (11.7%). Majority of them were treated with only 1 lipid-lowering agent. Using more than one lipid lowering agents was not common, and more US obese patients (4%) reported using at least 2 lipid lowering agents (0.7% in EU, 2.5% in CA, and 0% in AUS).

Table 4 shows gender differences in comorbid conditions. Across all obesity-related comorbidities, males were over-represented in HTN, T2DM, CVD and hyperlipidemia than females. However, there were no gender differences in disease duration or medication utilization patterns. Gender differences were not tested for statistical significance within each region due to small sample sizes for CA and AUS.

**Table 4:** Baseline Comorbidities by Gender.

	Overall (N = 1106)		Male (N = 229)	Female (N = 877)	P-Value
Hypertension, N (%)	474 (42.9%)		310 (49.5%)	99 (34.0%)	31 (39.2%)
Number of Years with Hypertension, Mean (sd)	7.7 (7.6)		8.4 (8.1)	7.4 (7.3)	.27
Hypertension Medication Usage, N (%)	391 (82.5%)		106 (83.5%)	285 (82.1%)	.79
Type 2 Diabetes, N (%)	245 (22.2%)		71 (31.0%)	174 (19.8%)	.001
Number of Years with Type 2 Diabetes, Mean (sd)					
Overall	7.0 (6.2)	6.4 (4.7)		7.2 (6.6)	.39
Patients treated with insulin	11.7 (6.9)	9.8 (3.8)		12.5 (7.8)	.18
Patients treated without insulin	5.5 (4.8)	5.0 (4.3)		5.6 (5.0)	.48
Anti-Diabetic Medication Usage, N (%)					
Treated with insulin	65 (26.5%)	19 (26.8%)		46 (26.4%)	.99
Treated with oral medication	186 (75.9%)	126 (72.4%)		126 (72.4%)	.05
Treated with diet	84 (34.3%)	19 (26.8%)		65 (37.4%)	.14
Without treatment	7 (2.9%)	0 (0%)		7 (4.0%)	.19
Unknown	1 (0.4%)	0 (0%)		1 (0.6%)	.99
Cardiovascular Disease, N (%)	49 (4.4%)	21 (9.2%)		28 (3.2%)	.0001
Number of Years with Any One of the Cardiovascular Diseases, Mean (sd)	6.2 (10.1)	9.5 (13.3)		3.5 (5.4)	.06
Hyperlipidemia Problem, N (%)	229 (20.7%)	77 (33.6%)		152 (17.4%)	.0001
Hyperlipidemia treated with lipid lowering agents, N (%)					
1 Med	200 (18.1%)	66 (28.8%)		134 (15.3%)	.59
≥2 Meds	29 (2.6%)	11 (4.8%)		18 (2.1%)	.66

Overall patient reported outcomes decreased as BMI increased by 5 kg/m^2^ increment (Table 5). Comparing BMI ≤35 and BMI >45 kg/m^2^, mean PCS score decreased from 46.7 (±7.00) to 37.2 (±11.50) and mean MCS score went from 47.0 (±10.78) to 41.2 (±10.14) between those lowest and highest categories. Similarly, comparing the lowest and highest categories of BMI, mean EQ-5D scores were 0.81 (±0.148) and 0.70 (±0.207) and IWQoL total scores were 64.1 (±16.37) and 41.5 (±20.19), respectively. Greater impairment for non-work related activities as measured by WPAI scores was also evident when the lowest and highest BMI categories were compared (41.4% v 55.2%) (Table 5).

**Table 5:** Baseline Patient Reported Outcomes by BMI.

Baseline BMI Categories (kg/m^2^)	SF-12, Mean (SD)		EQ-5D, Mean (SD)	IWQOL Total Score, Mean (SD)	WPAI % Non-Work Activity Impairment due to Health, Mean (SD)
	Physical Component Score	Mental Component Score			
≤35 (N = 14)	46.7 (7.00)	47.0 (10.78)	0.81 (0.148)	64.1 (16.37)	41.4 (33.25)
>35–≤40 (N = 241)	39.6 (10.91)	42.3 (9.99)	0.74 (0.180)	50.4 (18.03)	48.5 (25.73)
>40–≤45 (N = 389)	39.2 (11.09)	41.8 (9.40)	0.74 (0.174)	47.9 (18.64)	48.5 (26.85)
>45 (N = 462)	37.2 (11.50)	41.2 (10.14)	0.70 (0.207)	41.5 (20.19)	55.2 (28.98)

## Discussion

The HERO study is designed to provide long term outcomes of LBAP in “real-world” settings for a geographically diverse group of patients. Observational study designs like this are increasingly being used to report real world safety and outcomes data over longer term periods than what is often possible in randomized trials [28]. Further, HERO's broad eligibility criteria enhance its ability to provide descriptive information about the impact of heterogeneity on outcomes.

Strengths of the HERO study include prospective follow-up of its large patient pool, long-term follow-up on comorbid conditions, a multi-country approach which improves the generalizability of the data, use of validated instruments to assess HRQoL and productivity, and measure of associated resource utilization. The non-interventional design is also an asset since the study captures real-world behavior in the absence of protocol-driven treatments and behavioral supports [29]. However, this study also faces several challenges. A main practical challenge concerns retention of subjects over five years. In anticipation of this challenge, procedures have been developed for follow-up for key outcomes by telephone and mail for those who do not return to their surgeon (see Methods Section.) In addition, it may be difficult to distinguish whether the benefits and risks of the procedure can be disentangled from other support characteristics such as frequency of follow-up and nutritional counseling that contribute to successful long-term weight control with any surgical weight-loss procedure.

The demographic characteristic and comorbidity profile of the HERO cohort is representative of those who opt for the procedure as reported in the literature. Similar to the Nationwide Inpatient Survey and The Bariatric Outcomes Longitudinal Database (BOLD) registry and a meta-analysis of 612 international bariatric surgery studies, the HERO cohort is predominantly female (80%), with a mean age between 44–47 years [30]–[32]. A body mass trajectory study also revealed that weight gain is greater among women than men suggesting they may be at greater risk of obesity-related conditions without intensive weight management [33]. Women are also more likely to seek bariatric surgery than men, who tend to be more motivated by other concurrent medical conditions than weight outcomes [34].

Hypertension is the most common comorbidity in obesity, followed by diabetes. The overall prevalence of HTN in this study is also comparable to the 38 to 52% found in previous US and multinational studies among bariatric surgery patients, [31], [32], [35]–[38] although the Canadian Institute for Health Information Discharge Abstract Database in 2002–2003 reported a lower rate of 10% HTN and 10% diabetes among bariatric patients [39]. This is notable in that most of the patients are female and relatively young compared to the overall hypertensive population.

Interestingly, regional differences in the prevalence of baseline comorbidities were observed in the HERO study. US LBAP cohort reported larger proportions of T2DM, HTN, and CVD, as compared with the other three regions. This may be due to variations in comorbid conditions requirements for coverage across countries, or may be due to more aggressive use of surgical weight loss in the US for obese patients with comorbidities.

Baseline HRQoL results from HERO corroborate previous studies which have shown that the severely obese (BMI >40 kg/m^2^) reported worse general health as well as emotional and social impact compared to those with mild obesity (BMI 30–34.99 kg/m^2^) [40]–[44].

High-quality long-term evidence on the risks and benefits of the most up-to-date gastric banding devices and techniques will help surgeons and patients make informed decisions about the usefulness and desirability of this approach to control obesity and its related comorbidities [45]. The consistency of information, heterogeneity of patients, and long-term follow-up collected in the HERO study will provide a large resource of systematic naturalistic data on weight loss, changes in comorbidities, and patient reported outcomes.

Findings from HERO will assist patients and physicians in making better informed treatment decisions for obesity and its related comorbidities, as well as provide meaningful data from a robust and generalizable sample which should help to update standards of practice for the treatment of obesity. Overall, these data fill important gaps in knowledge about the effectiveness of current surgical techniques as practiced in real-world settings, both from the perspective of the physician and the patient. Additional research questions to pursue include, but are not limited to, regional differences in reduction of comorbidities over time and how those differences may affect HRQoL.
